# The natural history of curve behavior after brace removal in adolescent idiopathic scoliosis: a literature review

**DOI:** 10.1007/s43390-022-00638-x

**Published:** 2023-01-30

**Authors:** Scott Luhmann, Daphna Zaaroor-Regev, Vidyadhar V. Upasani, Harry Shufflebarger

**Affiliations:** 1 Washington University School of Medicine, 660 S Euclid Ave, St. Louis, MO 63110-1010 USA; 2 ApiFix Ltd., Yokneam Elit, Israel; 3 Department of Orthopaedic Surgery, Rady Children’s Hospital, San Diego, CA 92123 USA; 4 Paley Orthopedic and Spine Institute at St. Mary’s Medical Center, 901 45th Street, West Palm Beach, FL 33407 USA

**Keywords:** Adolescent idiopathic scoliosis, Brace treatment, Curve progression

## Abstract

**Purpose:**

Brace treatment is the most common nonoperative treatment to prevent curve progression in adolescent idiopathic scoliosis (AIS). The goal of this review and analysis is to characterize curve behavior after completion of brace treatment and to identify factors that may facilitate the estimation of long-term curve progression.

**Method:**

A review of the English language literature was completed using the MEDLINE (PUBMED) database of publications after 1990 until September 2020. Studies were included if they detailed a minimum of 1 year post-brace removal follow-up of AIS patients. Data retrieved from the articles included Cobb angle measurements of the major curves at “in-brace,” weaning, and follow-up visit(s) for all patients described and for subset populations.

**Results:**

From 75 articles, 18 relevant studies describing a follow-up period of 1–25 years following brace removal were included in the analyses. The reviewed literature demonstrates that curves continue to progress after brace treatment is completed with three main phases of progression: (i) immediate (upon brace removal) where a mean curve progression of 7° occurs; (ii) short term (within five years of brace removal) where a relatively high progression rate is evident (0.8°/year); and (iii) long term (more than five years after brace removal) where the progression rate slows (0.2°/year). The magnitude and rate of curve progression is mainly dependent on the degree of curve at weaning as curves weaned at < 25° progress substantially less than curves weaned at ≥ 25° at 25 years.

**Conclusion:**

Curves continue to progress after brace removal and the rate and magnitude of progression are associated with the curve size at weaning, with larger curves typically exhibiting more rapid and severe progression. This analysis provides physicians and patients the ability to estimate long-term curve size based on the curve size at the time of weaning.

**Level of evidence:**

IV.

**Supplementary Information:**

The online version contains supplementary material available at 10.1007/s43390-022-00638-x.

## Introduction

The prevalence of Adolescent idiopathic scoliosis (AIS) is estimated to be between 1 and 3% for children between 10 and 16 years of age and is more prevalent in girls than boys. The etiology for the condition is considered multifactorial with both intrinsic and extrinsic factors.

To date, the most extensive long-term observations of natural curve behavior over time is the series of Iowa studies that shaped our understanding of the natural history of AIS. Their data compilation teaches us that curves > 30° at skeletal maturity progress at a rate of < 1° per year. In other words, as the curve size increases, so does the likelihood of its progression [1]. The Iowa studies, although fundamental, detail the rate of progression from the time of diagnosis up to 50 years, without distinguishing between the curve progression rates at skeletal immaturity and maturity.

When patients are skeletally immature, curve progression is mostly dependent on the curve magnitude and skeletal maturity (i.e., Risser score and Sanders grade). The most frequently utilized treatment to halt curve progression when these patients fall below the surgical threshold (45°–50°) is brace treatment. The Scoliosis Research Society (SRS) recommends brace treatment in patients with AIS from around 20°–45° during growth and a regular follow-up to assess brace performance at least every 3–6 months [2, 3]. Literature suggests that brace treatment is most effective in skeletally immature children, Risser 0–2, with curves between 20º and 40º [4].

In the literature, Cobb angles are typically assessed at four timepoints during nonsurgical treatment:

- At the initiation of brace treatment
- During treatment (usually referred to as “in-brace correction”)
- At brace weaning
- At the last follow-up

The purpose of the current review was therefore to characterize curve behavior after the completion of brace treatment and identify factors that may facilitate the estimation of long-term curve progression based on the curve magnitude at weaning.

## Methods

A systematic literature search was done using the MEDLINE (PUBMED) database using the following search terms and strategy:

Search terms:

- ((adolescent idiopathic scoliosis) AND (Brace or bracing)) AND (removal or removing or wean or weaning)

Search filters:

- Exclude non-English language manuscripts
- Include publications after 1990

Inclusion criteria:

- Studies were included in the analysis if they included AIS patients, a minimum of 1-year post-weaning follow-up, and had a sample size of ≥ 15 patients.
- Articles were scanned for the presence of information of curve behavior from bracing to after brace removal. Articles that described both AIS and JIS were included; however, if the AIS population was described separately, it is reported in this review.

Exclusion criteria:

- Review publications and conference abstracts.
- Articles describing nighttime bracing only.

Two (2) researchers with many years of relevant experience and deep knowledge of the scoliosis field reviewed the titles and abstracts independently, and in cases of disagreement, a third reviewer was consulted.

Data retrieved from the articles included Cobb angle measurements of the major curves at “in-brace”, weaning, and follow-up visit(s) for all patients described and for subset populations, if available.

Where available, “in-brace” measurement descriptions (e.g., best in-brace, first in-brace, intermediate time) were noted. Where information was not detailed in the text, it was extracted from graphs, if available.

The population included in the present review was of patients weaned from brace at skeletal maturity. Brace weaning criteria for the relevant articles are detailed in Table 1.

**Table 1:** Summary table of articles included in the review

Author	Level of evidence	N	Brace weaning criteria	Last FU max. length [years]	Cobb Angle			Rate Delta (Last FU—Weaning) / FU time weighted mean
					In brace	Weaning	Last FU	
Aulisa et al. [20]	Therapeutic Level IV	40	Weaning was started when ring-apophysis fusion was seen to begin	3.5	8.4 ± 5.2	11.6 ± 7.7	13.8 ± 7.9	0.61
Aulisa et al. [21]	Therapeutic Level IV	50	Weaning was started when ring-apophysis fusion was seen to begin	4.6	12^b^	12	14.7 ± 7.6	0.97
Aulisa et al. [12]	Therapeutic Level IV	69	Weaning was started when ring-apophysis fusion was seen to begin	3.5	16.6 ± 9.0	16.3 ± 9.6	20 ± 7.6	1.06
Aulisa et al. [11]	Prognostic Level II	253 Complete compliance	Weaning was started when ring-apophysis fusion was seen to begin	3.5	12^b^	10	13.1 ± 10	3.36
		15 (subgroup A)		3.5	16^b^	16	20.8 ± 6.8	0.31
		31 (subgroup B)		3.5	18^b^	20	24.2 ± 8.0	0.56
		60 (subgroup C)		3.5	18^b^	18	20.1 ± 7.5	0.54
		36 (subgroup D)		3.5	19^b^	22	23.1 ± 9.0	0.17
Peltonen et al. [24]	Therapeutic Level IV	25 (subgroup TL)	Weaning started when the growth had ceased, after which the patients grad- ually gave up the brace during a 6-month period	3	21	31	36	0.56
		28 (subgroup L)			26	33	35	0.25
		49 (subgroup DM)			26	33	41	1.76
		60 (subgroup T)			22	27	33	1.61
Aulisa et al. [18]	Prognostic Level III	93	Weaning was started when ring-apophysis fusion was seen to begin	15.3		19.4 ± 10.8	22.1 ± 12.1	> 30 @ end of treatment 0.07
								> 30 at initiation 0.16
								≤ 30 @ end of treatment 0.25
								≤ 30 at initiation 0.12
Pellios et al. [10]	Prognostic Level III	77	Skeletal maturity de fined as a Risser sign ≥ 4 or the age of 16 and 18 years for girls and boys respectively. 3 years postmenarche	25.2	17.3 ± 9.2^a^	21.6 ± 11.5	25.5 ± 13.9	0.12
Guo et al. [7]	Therapeutic Level II	30 (subgroup stable group)	Skeletal maturity which was defined by < 1 cm change in standing height made on two consecutive visits with 6 months apart, when Risser 4 was present and when the patient was 2-year postmenarche	4	19.4	21.8 ± 6	27 ± 7	1.38
Basset et al. [23]	Therapeutic Level IV	16 (Subgroup DM	Weaning was started when iliac apophysis is shown to be fully capped (Risser 4)	2.5	18^a^	31	33	0.08
		24 (Subgroup TL			12^a^	22	23	0.09
		39 (Subgroup T			14^a^	29	31	0.12
Brox et al. [14]	Therapeutic Level IV	274 (Subgroup Compliers, long term)	Either 2 years after menarche or at Risser sign 5, in some patients at Risser sign 4	23.5	16 ± 6	26 ± 9	32 ± 14	1.29
		54 (Subgroup Non-compliers, long term)		23.5	17 ± 6	31 ± 10	38 ± 10	0.23
Lang et al. [9] (subset of Brox et al. [14])	Therapeutic Level IV	86	Not detailed, however, age at weaning was 16.2 ± 1.1 years	19.2	NA	28.3 ± 9	34.2° ± 14	0.45
Lange et al. [15]	Therapeutic Level IV	215 (subgroup Curve < 45° at last follow-up)	Either 2 years after menarche or at Risser sign 5, in some patients at Risser sign 4	24.7	15 ± 6	25.1° ± 8.2°	29.2° ± 9.4°	0.38
		32 (Subgroup Curve ≥ 45° at last follow-up)		24.7	21 ± 6	37.3° ± 7.0°	55.0° ± 8.7°	0.25
Montgomery et al. [8]	Prognostic Level III	168	Not detailed, however, age at weaning was 16.0 ± 2.2 years	6.9	NA	28.0 ± 10.7	33.1 ± 11.5	1.50
Danielsson and Nachemson [19]	Prognostic Level III	109	Risser degree of 4 or 16 years of age for girls and 18 years of age for boys	22.2	24.7 ± 10.9^a^	29.7 ± 11.2	37.6 ± 14.7	0.37
Shi et al. [17]	Therapeutic Level IV	200	Risser stage > 4 and more than 2 years postmenarche and 2) no growth between 2 visits	4.3	21.7 ± 7.6^a^	30.1 ± 10.4	35.6 ± 12	2.97
Cheung et al. [16]	Prognostic Level III	144	Risser Stage 4, had no growth in body height, sitting height, and armspan in the past 6 months offollowup, andwere at least 2 years postmenarche	3	NA	35	43 ± 3	5.16
Korovessis et al. [22]	Level III	43 (Subgroup TL)	treatment for at least three years. Bracing was terminated mainly because of skeletal maturity (Risser V)	2.6	24 ± 6^c^	NA	23.4 ± 7.6	NA
		(Subgroup L)			25 ± 12^c^	NA	25.2 ± 10.5	NA
		(Subgroup T)			29 ± 10^c^	NA	31.3 ± 7.2	NA
Cheung et al. [6]	Therapeutic Level III	98 (Subgroup Improved)	Risser stage 4, no growth in body height for the past 6 months, and 2 years postmenarche	2	14^c^	22	22	0.0
		234 (Deterioration Improved)		2	20^c^	46	46	0.0
		254 Unchanged		2	17^c^	30	31	20.15

^a^Best in-brace

^b^Intermediate time

^c^First in brace

### Statistical considerations

The review was conducted from 18 papers. Statistical analysis was performed by a Senior Biostatistician (M.Sc.). The data collected were the follow-up time and three measurements of Cobb angle as available: in brace, in weaning, and at follow-up. In-brace Cobb angle measurement represented the curve’s size at the beginning of the weaning process. Where relevant, data is presented as mean ± standard deviation (SD). When only the range was available, SD was calculated as Range/4 [5].

When there was no information on variability, the SD was extrapolated using the mean of the SD of other papers. The SD of the changes were calculated as

$${SD}_{\mathrm{change}}=\sqrt{{SD}_{\mathrm{baseline}}^{2}+{SD}_{\mathrm{final}}^{2}-(2\times {\mathrm{corr}}_{\mathrm{baseline}-\mathrm{final}}\times {\mathrm{SD}}_{\mathrm{baseline}}\times {\mathrm{SD}}_{\mathrm{final}})}$$

where relevant, the change from baseline (weighted) was calculated by imputed SD using the imputed correlation coefficient, where the correlation coefficient ${\mathrm{corr}}_{\mathrm{baseline}-\mathrm{final}}$ was extrapolated using the full set of papers. A similar method with correlation coefficients was used for the SD of the rates. Each analysis was based on the generic inverse variance method for meta-analysis. Specifically, each subgroup’s weighted average was calculated as a comparison between subgroups, which were applied using a chi-square test for heterogeneity across subgroups. Statistical significance for the trend in Fig. 2 was taken from a test of the slope in a weighted regression using

$$\frac{\sum {\mathrm{Mean}}_{i}\times \left(\frac{1}{{\mathrm{Standard\, Error}}_{i}^{2}}\right) }{\sum \frac{1}{{\mathrm{Standard\, Error}}_{i}^{2}}}$$

## Results

**Fig. 1:**
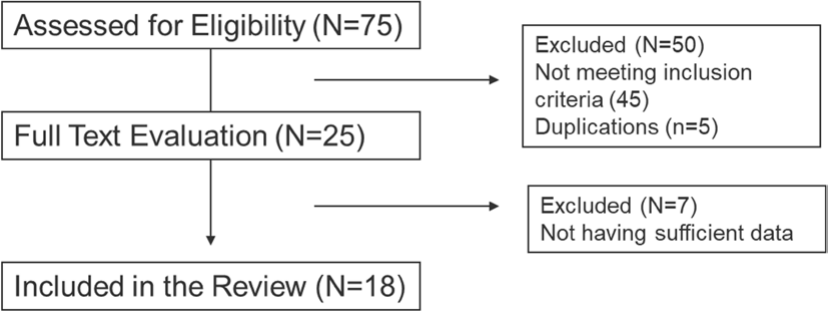
CONSORT type flow diagram of the article selection process

A total of 75 articles were identified in the Medline database using the search terms and strategy. The data encompassing 2107 AIS patients over a follow-up period of 1–25 years post-brace removal were reviewed and summarized (Fig. 1).

Most articles assessed the population as a whole, whereas some also subdivided the findings in relation to curve type, compliance with brace wear, and curve magnitude. Most publications followed the accepted guidelines for brace treatment (10–12 years of age, skeletally immature with curves Cobb angle ranging largely between 20° and 40°) and brace weaning (skeletal maturity and no growth over some time, taking into consideration time lapse since menarche).

i. Studies assessing the population as a whole

Cheung et al. [6] retrospectively studied 586 patients with 2.0 ± 1.1 years follow-up post-brace weaning. Participants were classified by their response to pre-brace to weaning as improved, unchanged, or deteriorated. During the time from in-brace to weaning, curves continued to progress in the 3 groups: from 14° to 22°, 17°–30°, and 20°–45°, respectively. Curve deterioration was experienced by 40% of patients, for whom the mean first-in-brace, weaning, and last follow-up Cobb angles were 20°, 45°, and 46°, respectively, with a progression rate of 0.5°/year.

Guo et al. [7] investigated curve behavior in patients treated with a rigid brace or a SpineCor brace. In-brace, weaning, and last follow-up curve details were provided for 30 participants whose results were termed “Stable”: 17 patients treated with a rigid brace (R-stable group) and 13 treated with a SpineCor brace (S-stable group). The mean first-in-brace, weaning, and last follow-up Cobb angles of the R-stable group were 20.0°, 21.8°, and 27.4°, respectively, and 18.9°, 21.7°, and 27.7° for the S-stable group. Between weaning and the last follow-up (R-stable: 47.4 ± 14.9 months; S-group: 43.1 ± 11.9 months), each group experienced an average increase of 5.2°, representing a yearly rate of 1.3°. The proportions of patients who had curve progression > 5° after weaning were 29.4% in the R-stable group and 38.5% in the S-stable group.

Montgomery et al. [8] followed 168 patients for 6.9 ± 2.5 years after brace removal. Mean curve magnitudes were 28.0° ± 10.7° at brace weaning and 33.1° ± 11.5° at last follow-up. Most progression occurred within two years of brace removal (4.5°, yearly rate of 2.2°). An additional 0.6° progression occurred by the last follow-up, for a total of 5.1° from weaning to last follow-up. The proportion of participants who progressed ≥ 7.5° between weaning and last follow-up was 30%.

Lange et al. [9] followed 86 patients for a mean of 19.2 years after brace removal. Mean primary curves measured 28.3° (range 9°–53°) at brace weaning and 34.2° (range 8°–87°) at final follow-up. Interestingly, on average, at the last follow-up, the primary curve progressed to its size as at the start of bracing at a rate of 0.3° per year. Eleven patients (12.7%) had a major curve measuring > 45° at the last follow-up: in 6, the curve exceeded 60°, and in 2, the curve exceeded 85°. The mean progression between weaning and last follow-up was 22.5°.

Pellios et al. [10] evaluated curve behavior in 77 patients with a mean follow-up of 25 years after brace weaning. Mean primary curves measured 17.3° ± 9.2° at first in-brace measurement, 21.6° ± 11.5° at weaning, and 25.5° ± 13.9° at last follow-up. Between weaning and last follow-up, the average increase was 3.9° ± 6.7°, representing a yearly rate of 0.2°. The proportions of patients with curves < 30°, 30°-40°, and > 40° at last follow-up were 71.4%, 18.2%, and 10.4%, respectively. Overall, 35% of the cohort had > 5° progression.

Data from the five studies assessing the population as a whole demonstrate that after brace removal, the yearly progression rate ranges from 0.2°/year to 2.2°/year, and the majority of overall progression during skeletal maturity occurs in the initial years after brace weaning.

ii. Impact of brace-wear compliance

Aulisa et al. [11] studied 367 patients from pre-brace treatment to a minimum of 2-years after brace weaning and described clinical outcomes relative to 3 groups of brace-wear compliance: (1) complete, (2) did not wear for up to 2 months per year, or (3) night-wear only or removal during school hours. The mean total follow-up was not reported, but we estimated it as 3.5 years based on information published in a subsequent article [12]. Cobb angle reduction during brace treatment depended on brace-wear compliance, which agrees with a previous review [13]. However, curve progression after brace weaning occurred in all groups. Mean curve progressions and annual rates for each group were (1) 3.1° and 0.9°/year, (2) 4.2°–4.8° and 1.2°–1.4°/year, and (3) 1.1°–2.1° and 0.3°–0.6°/year.

Brox et al. [14] investigated the relationship between curve behavior after brace removal and brace-wear compliance in idiopathic scoliosis (IS) patients. At weaning, compliant (*n = *384) and non-compliant patients (*n = *106) had mean curve magnitudes of 26.4° ± 9.5° and 33.5° ± 10.2°, respectively. At the 2-year follow-up, the curve magnitudes were 28.1° ± 9.2° and 33.2° ± 9.9° in compliers (*n = *355) and non-compliers (*n = *82), respectively. However, at the last follow-up (23.5 ± 4 years), 24% (68/284) of compliers and 65% (46/71) of non-compliers had curve progressions of ≥ 6°, although the mean overall progression and annual progression rate of the two groups were similar: 5° vs. 6° and 0.2°/year vs. 0.3°/year.

Neither of the included studies found a relationship between brace-wear compliance and curve progression after brace removal.

iii. Impact of curve size

Lange et al. [15] studied 247 late-onset juvenile scoliosis and AIS patients from in-brace treatment to a mean last follow-up 24.7 years post-brace treatment. Patients were categorized into two groups by the Cobb angle at the last follow-up: < 45° (*n = *215) or ≥ 45° (*n = *32). For the < 45° group, average Cobb angles in-brace, at weaning, and at the last follow-up were 15°, 25.1° ± 8.2°, and 29.2° ± 9.4°, respectively (mean curve progression of 4° and rate of 0.2°/year), whereas the same measurements in the > 45º group were 21°, 37.3° ± 7.0°, and 55.0° ± 8.1° (mean curve progression of 17.7° rate of 0.7°/year).

Cheung et al. [16] studied 144 patients from brace weaning to a mean follow-up of 3.0 ± 1.7 years. The average Cobb angle at weaning was 35° with a mean curve progression of 8.3° ± 3° and rate of 2.7°/year. The proportions of patients with curves < 45° at weaning and those with curves ≥ 45° who experienced > 5° of progression at last follow-up were 25% and 62.5%, respectively. The authors concluded that a Cobb angle of ≥ 45° at brace weaning is an independent risk factor for progression after brace weaning (*p = *0.002).

Shi et al. [17] studied 200 girls from brace weaning to a mean follow-up 51.4 ± 25.6 months. The mean Cobb angles at weaning and last follow-up were 30.1° ± 10.4° and 35.6° ± 12.0°, respectively. At brace weaning, 13% (*n = *26) had a Cobb angle of > 40°, and the curves of these patients progressed to a mean of 52.8° ± 9.1° at last follow-up. Among the 174 patients with Cobb angles ≤ 40° at weaning, the proportions who experienced curve progression > 5° or a Cobb angle of > 45° at last follow-up were 43.5% or 10.3%, respectively. Comparisons of the at-weaning Cobb angles of patients above and below the thresholds of 5° curve progression or a Cobb angle of 45° at last follow-up revealed significantly higher at-weaning Cobb angles for patients above either threshold (*p < *0.001). In other words, patients with higher Cobb angles at weaning experienced higher magnitudes of curve progressions post-weaning.

Aulisa et al. [18] followed 93 patients from pre-bracing to a mean follow-up after brace removal of 15.3 ± 5.2 years. Overall, the study population had mean pre-brace, at-weaning, and 5- and 10-years post-weaning Cobb angles of 32.28º ± 9.4º, 19.4º ± 10.8º, 20.67 º ± 11.2º and 22.1º ± 12.1º, respectively, with no significant curve progression post-weaning (*p = *0.105). Long-term outcomes were compared between patients with at-weaning Cobb angles of ≤ 30° or > 30º. Patients in the > 30° group had a mean pre-brace, at-weaning, and final follow-up scoliotic curves of 43.94°, 34.89°, and 38.39°, respectively. In contrast, these values in the ≤ 30° group were 29.35°, 15.05°, and 18.21°. Although the difference in the at-weaning to final follow-up angle increases between the > 30° (3.5°) and ≤ 30° (3.16°) groups was not statistically significant, the magnitude of the curves at last follow-up has clinical significance (38.4° vs. 18.2°).

These articles point to an association between Cobb angle at weaning and magnitude of curve progression.

**Fig. 2:**
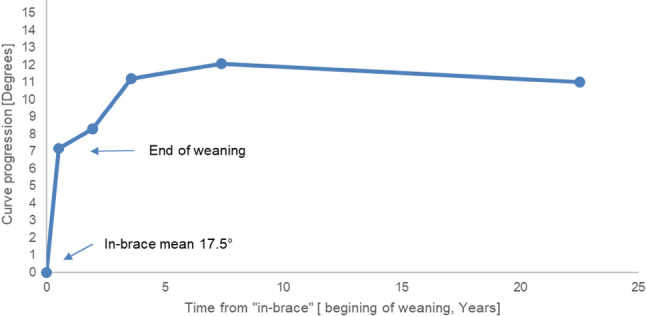
Mean curve progression over time. Average change in curve progression over time from “in-brace” to last available follow-up after brace removal

**Fig. 3:**
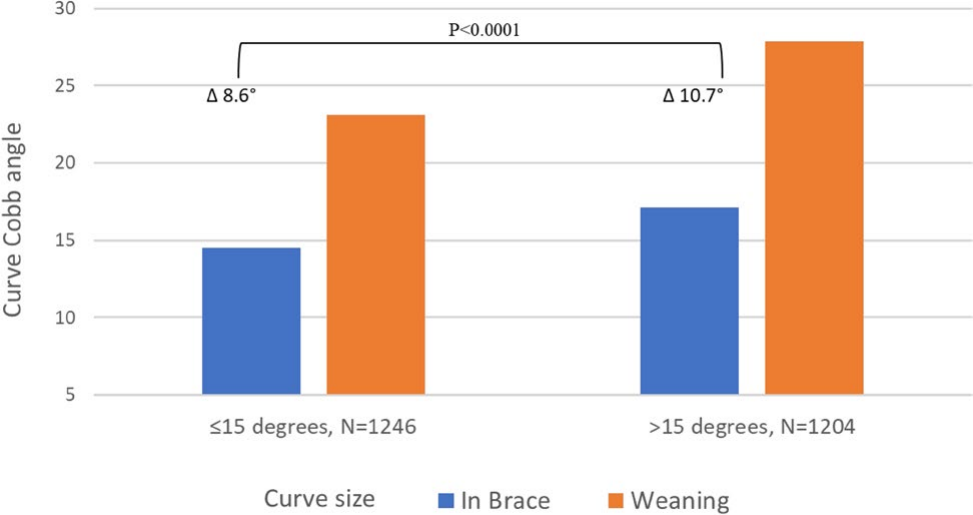
Dependency of mean curve progression from in-brace to end of weaning on curve size in-brace. Curve progression during brace wear in curves measured above or equal and below 15° “in-brace”. Average “in-brace” curve Cobb angle is presented in blue and average weaning Cobb angle is presented in orange

Data from the retrieved articles were pooled. The “in-brace” data point was considered a reliable indicator of curve size at the beginning of weaning (excluding data points identified as “best in brace” and “first in-brace”). Figure 2 provides an overview of the average curve progression. As can be seen, during the weaning process, an average increase of 7° is evident by the end of weaning, reaching 11° by the last follow-up. On average, curves reaching 17.5° at the beginning of the weaning process are estimated to stabilize and reach 28.5° in the long term. Figure 3 shows that curves reduced to ≤ 15° in-brace show less progression immediately after brace removal compared to curves > 15° in-brace (8.6° vs. 10.7°, *p < *0.001).

**Fig. 4:**
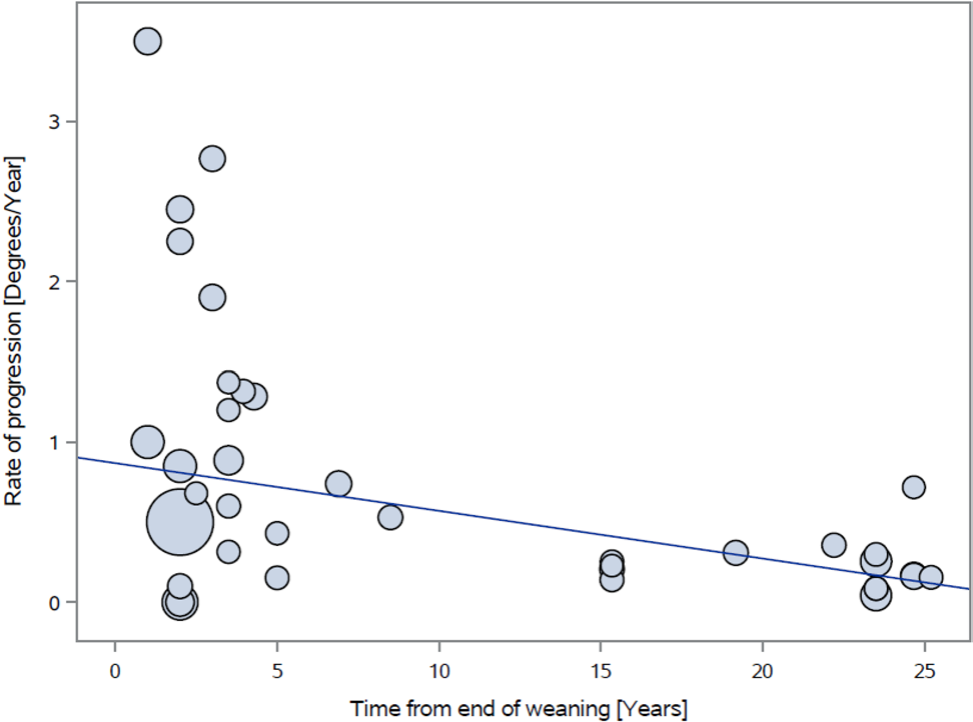
Rate of progression over time (from end of weaning to the last follow-up)

The progression rate from weaning to the last follow-up is described in Fig. 4. The data scatter pattern shows that in the short term (within five years of brace removal), the rate of curve progression is highly variable, and over time the variation becomes less. Nevertheless, from the end of weaning to the last follow-up, the rate of progression slows over time. Analysis shows that in the short term, curves progress at a higher rate than in the long term (0.8°/year vs. 0.2°/year *p = *0.039), with an overall progression rate of 0.7°/year.

Due to the comparatively small number of articles with data for weaning at 10º–15º and 45º–50º, analysis was performed based on a cutoff point of < 25º and ≥ 25º at weaning.

Curves weaned at < 25° (average 18.3°) measured on average 20° at last follow-up whereas curves weaned at ≥ 25° (average 29.7°) measured 35.7° at last follow-up (*p < *0.001).

**Fig. 5:**
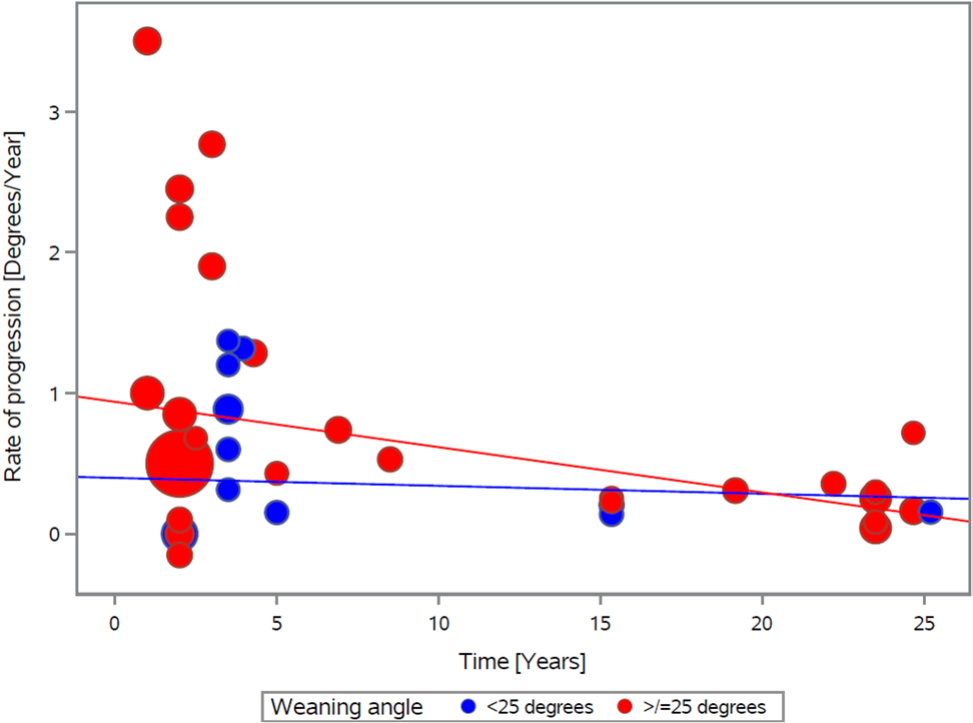
Curve progression rate from end of weaning to the last follow-up in dependence of curve size at end of weaning. Curve progression rate until last follow-up for curves weaned from bracing at a Cobb angle of equal and above (red) and below (blue) 25°

Analysis of the rate of curve progression from the end of weaning to the last follow-up in relation to curve size at the end of weaning is detailed in Fig. 5. Curves weaned at < 25° progress slower (0.4°/year) than curves weaned at ≥ 25° (0.7°/year); however, the difference was not statistically significant (*p = *0.101).

iv. Impact of curve type

Danielsson and Nachemson [19] studied 109 patients from before treatment to a mean of 22.2 ± 1.8 years after brace removal. Mean best-in-brace, at-weaning, 7–10 years follow-up, and last follow-up Cobb angles were 24.7° ± 10.9°, 29.7° ± 11.2°, 34.2° ± 12.8°, and 37.6° ± 14.7°, respectively. The curve progressions, following brace removal, at 7–10 years follow-up and last follow-up were 4.5° and 7.9°, the annual rates were 0.6°/year and 0.4°/year, respectively. The proportions of patients who experienced curve progressions of > 10° and > 20° at final follow-up were 36% (*n = *39) and 4.6% (*n = *5), respectively. A subgroup analysis by curve type showed that double-major curves progressed the most between weaning and last follow-up (*n = *40, 9.3°), followed by thoracic (*n = *47, 7.8°), thoracolumbar (*n = *18, 6.4°), and lumbar curves (*n = *4, 1°); however, the data for lumbar curves might be due to the small sample size.

Aulisa et al. [20] studied 40 patients with idiopathic lumbar scoliosis from pretreatment to a mean of 3.5 ± 2.8 years follow-up after brace removal. Mean in-brace, at-weaning, and last follow-up Cobb angles were 8.4° ± 5.2°, 11.6° ± 7.7°, and 13.8° ± 8.0°, respectively. The mean curve progression during the weaning process and the annual progression rate post-weaning were 3.2° and 2.2° and 0.6°/year, respectively.

In another study, Aulisa et al. [21] evaluated the thoracolumbar curve behavior in 50 patients from pretreatment to a mean follow-up of 4.6 ± 3.7 years after brace weaning. Mean in-brace, at-weaning, and last follow-up Cobb angles were 12°, 12°, and 14.7° ± 7.6°. The mean curve progression and annual rate post-weaning were 2.7° and 0.6°/year, respectively. No patient presented with a curve progression of ≥ 6° after treatment.

Aulisa et al. [12] studied 69 patients with thoracic idiopathic scoliosis from pretreatment to a mean follow-up of 3.5 ± 2.6 years after brace weaning. Mean in-brace, at weaning, and last follow-up Cobb angles were 16.6° ± 9.0°, 16.3° ± 9.6°, and 20° ± 7.6º. The mean curve progression and annual rate post-weaning were 3.7° and 1°/year, respectively.

Altogether, Aulisa et al. published three articles evaluating lumbar, thoracolumbar, and thoracic curve behaviors with a follow-up period of 3.5–4.6 years post-brace removal. Although the populations had relatively low mean Cobb angles at weaning (11.6°-16.3°), mean curve progressions after weaning ranged from 2.2° to 3.7° [12, 20, 21].

Korovessis et al. [22] followed 43 patients from pretreatment to a mean follow-up of 2.6 ± 0.6 years post-brace weaning. Mean in-brace measurements were performed at 1 month and 1 and 3 years of brace wear (at-weaning data was not collected); however, mean Cobb angles in-brace and at last follow-up were not significantly different. By curve type, mean curve progressions between 3 year in-brace and last follow-up for thoracic, thoracolumbar, and lumbar curves were 2.3°, -0.6°, and -0.2°.

Basset et al. [23] studied 79 patients affecting the thoracic (*n = *39), thoracolumbar (*n = *24), and double major (*n = *16) curves from pretreatment to a mean follow-up of 2.5 years (range 1–9). Mean Cobb angles for best-in-brace, at-weaning, and last follow-up for double-major curves were 18°, 31°, and 33°, respectively; for thoracolumbar curves, 12°, 22°, and 23°, and for thoracic curves, 14°, 29°, and 31°. Mean curve progressions and annual rate post-weaning ranged from 1° to 2° and from 0.4°/year to 0.8°/year, respectively. The proportions of patients with thoracic, thoracolumbar, or a double-major curve that progressed between pretreatment and last follow-up (progression from weaning to follow-up was not reported) were 35.8%, 16.7%, and 56.3%, respectively.

Peltonen et al. [24] followed 162 patients affecting the thoracic (*n = *60), thoracolumbar (*n = *25), lumbar (*n = *28) and double curves (*n = *49) from pretreatment to a mean follow-up of 3 years follow-up (range 1.5–7). Mean Cobb angles for in-brace, weaning, and last follow-up for thoracic curves were 22°, 27°, and 33°, respectively; for thoracolumbar curves, 21°, 31°, and 36°; for lumbar curves, 26°, 33°, and 35°, and for double curves, 26°, 33°, and 41°. Mean curve progression and annual rate between at-weaning and final follow-up were highest for double curves (8° and 2.7°/year, respectively), followed by thoracic (6° and 2°/year), thoracolumbar (5° and 1.7°/year), and lumbar curves (2° and 0.7°/year). In 22% of the study population, curves progressed > 5°.

These articles indicate that double-major curves progress the most over time, followed by thoracic, thoracolumbar, and lumbar curves.

**Fig. 6:**
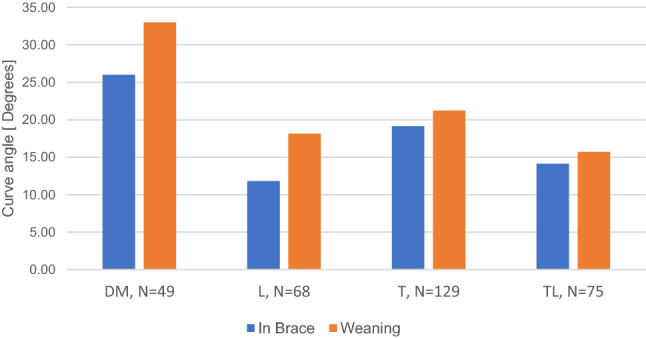
Progression from in-brace to end of weaning (DM, double major; T, thoracic; TL, thoracolumbar; L, lumbar; N, sample size)

The analysis of the above findings is presented in Fig. 6 and shows that the immediate curve progression (between in-brace and weaning) was highest for double-major curves, followed by lumbar thoracic and thoracolumbar curves (*p < *0.001). The analysis excluded data points identified as “best in brace and “first in-brace.”

**Fig. 7:**
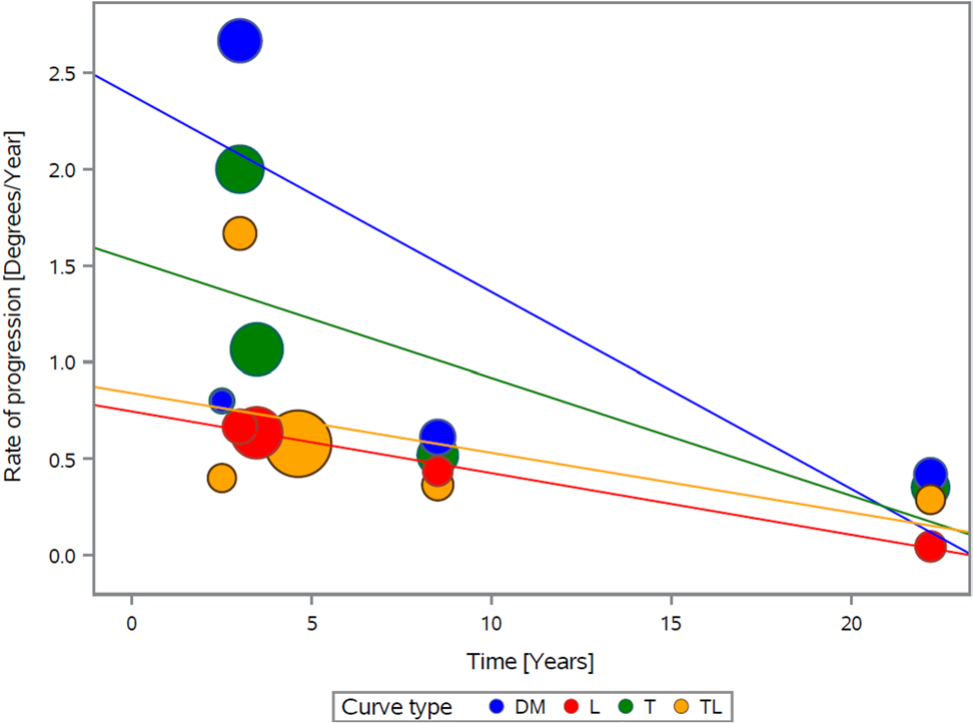
Rate of progression over time per curve type (from weaning to the last follow-up)

An evaluation of progression rate from the end of weaning to the last follow-up per curve type (Fig. 7) reiterated the same theme identified in Figs. 3 and 5: namely, that over time, the rate of curve progression slows. Short-term (within five years of brace removal), long-term, and overall annual progression rates were highest in double major (2.4°/year, 0.5°/year, and 1.49°/year, respectively), followed by thoracic curves (1.4°/year, 0.4°/year, and 1.1°/year), thoracolumbar curves (0.72°/year, 0.33°/year, and 0.65°/year), and lumbar curves (0.6°/year, 0.2°/year, and 0.5°/year). The differences in short term progression rates between groups were not statistically significant although they were clinically relevant.

It should be noted that the high progression rate for double-major curves might be a result of less efficient brace correction. On average, double-major curves that reached 32.5° at weaning progressed to an average of 39.7° at last follow-up. Conversely, thoracic, thoracolumbar, and lumbar curves typically reached < 25º at weaning (23.6°, 19.2°, and 19.0°, respectively), and then progressed to 27°, 22.5°, and 21.4° at last follow-up. The change from weaning to last follow-up weighted by imputed SD was statistically significant (*p < *0.001), which agrees with the finding reported earlier that curves weaned at < 25° progress less than curves weaned at ≥ 25°.

## Discussion

This literature review and analysis summarized the findings of 18 studies on patients with AIS who completed brace treatment and had at least one year of follow-up after brace removal (range: 1–25 years) to characterize post-treatment curve behavior in skeletally mature patients and identify factors that influence long-term progression, based on curve magnitude at brace weaning.

This review demonstrates that curves continue to progress in skeletally mature individuals after brace treatment is completed. However, the progression rate is slower than that of skeletally immature patients, who have a more rapid curve progression rate [25].

Our analysis identified an overall progression rate of 0.7°/year from weaning to last follow-up for the entire pooled sample. An in-depth analysis identified three phases of progression: immediate (upon brace removal) where a mean progression of 7° in the scoliotic curve occurs, short term (within five years of brace removal) where a relatively high progression rate is evident (0.8°/year), and long-term (more than five years after brace removal), where the progression rate slows (0.2°/year).

Patients whose curves experienced in-brace corrections to ≤ 15° had less substantial immediate progression than patients whose curves were corrected to > 15° (8.6° vs. 10.7°, respectively; *p < *0.001).

Notably, curves weaned at < 25° progress less substantially than curves weaned at ≥ 25°, reaching means of 20° vs. 35.7° at last follow-up. The difference between curves weaned at < 25° and curves weaned at ≥ 25° weighted by imputed SD was statistically significant (*p < *0.001). The progression rate is also more favorable with smaller curves at weaning because curves < 25° progressed at 0.4°/year vs. 0.7°/year for curves ≥ 25°. Although this finding failed to reach statistical significance, it has clinical value.

The included articles also permitted subgroup analyses to investigate the impacts of curve type and brace-wear compliance on curve progression.

The analysis of curve behavior after brace removal in relation to curve type showed that in-brace correction varied. Double-major, thoracic, thoracolumbar, and lumbar curves achieved average in-brace curves of 26°, 19.1°, 14.1°, and 11.8°, respectively. Not surprisingly, the curves at weaning varied with a similar trend: double-major curves had the highest Cobb angles (mean, 33°), followed by thoracic (23.6°), thoracolumbar (19.2°), and lumbar (19°). Similarly, double-major curves progressed at the highest rate (short term, 2.4°/year; long-term, 0.5°/year), followed by thoracic curves (short term, 1.4°/year; long-term, 0.4°/year), thoracolumbar (short term, 0.7°/year; long-term, 0.3°/year), and lumbar (short term, 0.6°/year; long-term, 0.2°/year). Although the differences in progression rates failed to reach statistical significance, our findings imply that different curve types do not behave differently due to their anatomical location. Instead, they follow the general theme identified in this work: less effective in-brace correction results in more rapid progression at weaning and final follow-up.

Ascani et al. [26] followed 187 untreated idiopathic scoliosis patients for 15–47 years after skeletal maturity. They reported that the greater the curve at maturity, the greater its progression, consistent with our findings. Overall, curves progressed on average 0.4°/year in Ascani et al. We found an overall progression rate of 0.7°/year, which is a reasonable difference. Ascani et al. [26] also reported that progression magnitudes vary with curve type: thoracic curves progressed the most, followed by lumbar, thoracolumbar, and double-major curves. They also reported that curves ≥ 40° progressed more than curves < 40°. We identified a different progression profile where double-major curves progressed the most, followed by thoracic, thoracolumbar, and lumbar. The difference between studies may be attributed to variation in the numbers of patients in each curve type group and that untreated patients reach skeletal maturity with a higher Cobb angle than brace-treated patients.

Lastly, our findings show that brace-wear compliance per-se was not associated with curve progression after brace removal. However, the data reviewed in this article may be insufficient to accurately assess brace-wear compliance and its impact on curve progression due to the longitudinal nature of this treatment process in a population that has been challenging to monitor over such a long time. Developing methods to monitor brace-wear compliance as a variable may increase our understanding of its effects on long-term outcomes because longitudinal surveillance is more feasible now than in the past.

Granular information regarding the extent of rotation, brace-weaning criteria, length of follow-up, and the degree of deformity at the pre-brace, in-brace, at weaning, and last follow-up stages are provided in the supplementary data table and Table 1 of the article.

Except for rotation, which was reported in only half (9 of 18) of the articles reviewed, all other granular information was analyzed in this review. The authors do agree that the degree of rotation warrants investigation; however, a more extensive literature search is required to increase the studied sample size.

Although our literature search was extensive, this review has some limitations. We searched a single database (MEDLINE) and excluded articles not published in English. Brace-wear protocols were relatively consistent across the articles included in this analysis, whereas weaning protocols demonstrated more variability. In addition, the timing of in-brace data was not clearly defined in all articles (e.g., whether the measurements represented first-in-brace or an intermediate time point). We excluded from specific analysis articles that defined in-brace data as “best in brace” or “first in-brace.” To that end, it is essential to bear in mind that all values given here are weighted averages, and a significant personal variation can be expected around these numbers.

## Conclusion

The presented literature review improves our understanding of how curves continue to progress after brace removal and the expected progression rate. Specifically, curve progression is most rapid during brace weaning and then slows to a relatively moderate pace within five years of brace removal (short-term phase); beyond five years (long-term phase), progression becomes minimal.

A better understanding of curve progression risk and rate during different phases after brace removal may allow physicians to estimate the magnitude of a patient’s curve in the future, based on the curve type and size at weaning.

In addition, this review supports the general theme that less effective in-brace correction results in more rapid progression at weaning and final follow-up. This theme emphasizes the long-term impact of corrective interventions before skeletal maturity. It provides a means to compare the effectiveness of treatments, resulting in better patient care and earlier, more accurate estimations of curve progression.

## Supplementary Information

Below is the link to the electronic supplementary material.

Supplementary file1 (DOCX 51 kb)

## Data Availability

The data used to support the findings of this study are available from the corresponding author upon request.
