# Supplementary material for: The natural history of curve behavior after brace removal in adolescent idiopathic scoliosis: a literature review
Source: Spine Deform. 2023 Jan 30;11(3):567–78. doi: 10.1007/s43390-022-00638-x (PMC10147768; doi:10.1007/s43390-022-00638-x)
Supplement: Supplementary file 1 — Supplementary file1 (DOCX 51 kb) [file 43390_2022_638_MOESM1_ESM.docx]

Supplementary data

| Author | N | Cobb Angle | | | | Degree of Rotation | | % of patients surgically treated |
| --- | --- | --- | --- | --- | --- | --- | --- | --- |
|  |  | Pre-brace | In brace | Weaning | Last FU | Baseline | End of treatment |  |
| **Aulisa et al**., [20] | 40 | 26.4 ± 2.8 | 8.4 ± 5.2 | 11.6 ± 7.7 | 13.8 ± 7.9 | 10.8±3.7 | 7.9±4.2 | NA |
| **Aulisa et al.,**[21] | 50 | 29.30 ± 5.16 | 12** | 12 | 14.7 ± 7.6 | 12.70 ± 6.14 | 8.95 ± 5.82 | 0 |
| **Aulisa et al**.,[12] | 69 | 31.51 ± 4.34 | 16.6 ± 9.0 | 16.3± 9.6 | 20 ± 7.6 | 13.09 ± 3.56 | 9.21 ± 4.5 | 1.4 |
| **Aulisa et al**.,[11] | 253 Complete compliance | 30.5 ± 8 | 12** | 10 | 13.1±10 | 13.2 ± 5.6 | 7.6 ± 4.8 | NA for the AIS population, for the entire sample 0.9% |
|  | 15 (subgroup A) | 30.5 ± 5.1 | 16** | 16 | 20.8 ±6.8 |  |  | NA for the AIS population, for the entire sample 3.7% |
|  | 31 (subgroup B) | 28.6°± 7° | 18** | 20 | 24.2±8.0 |  |  | NA for the AIS population, for the entire sample 7.1% |
|  | 60 (subgroup C) | 30.2 ± 6.7 | 18** | 18 | 20.1± 7.5 |  |  | NA for the AIS population, for the entire sample 2.7% |
|  | 36 (subgroup D) | 31.5 ± 8.8 | 19** | 22 | 23.1± 9.0 |  |  | NA for the AIS population, for the entire sample 8.7% |
| **Peltonen et al**.,[24] | 25 (subgroup TL) | 36 | 21 | 31 | 36 | Rotation of the apical vertebra correlated with the degree of the scoliotic curve and remained unchanged before and after the treatment | | 1.8% |
|  | 28 (subgroup L) | 37 | 26 | 33 | 35 |  |  |  |
|  | 49 (subgroup DM) | 40 | 26 | 33 | 41 |  |  |  |
|  | 60 (subgroup T) | 35 | 22 | 27 | 33 |  |  |  |
| **Aulisa et al**., .[18] | 93 | 32.28±9.4 |  | 19.4±10.8 | 22.1±12.1 | 13.86 (± 5.04) | 10.4 ± 6.2 | NA |
| **Pellios et al**.,[10] | 77 | 28.2±8.7 | 17.3± 9.2* | 21.6± 11.5 | 25.5± 13.9 | 15.09±6.9-13.69±6.48 | 13.66±9.03-14.86±9.01  At brace removal | NA |
| **Guo et al**.,[7] | 30 (subgroup stable group) | 24±2.9 | 19.4 | 21.8±6 | 27±7 | NA | NA | 6.7% |
| **Basset et al**., [23] | 16 (Subgroup DM | 30 | 18* | 31 | 33 | NA | NA | 12.5% |
|  | 24 (Subgroup TL | 25 | 12* | 22 | 23 |  |  | 0 |
|  | 39 (Subgroup T | 29 | 14* | 29 | 31 |  |  | 18% |
| **Brox et al**.,[14] | 274 (Subgroup Compliers, long term) | 33 | 16±6 | 26±9 | 32±14 | NA | NA | 3.5% in the entire Compliers, long term cohort n=284 |
|  | 54 (Subgroup Non-compliers, long term) | 32 | 17±6 | 31±10 | 38±10 |  |  | 24% in the entire Compliers, Non-compliers, long term n=71 |
| **Lang et al**.,[9] (subset of **Brox et al**.,[14] ) | 86 | 33.4 | NA | 28.3±9 | 34.2° ±14 | NA | NA | 6.5% |
| **Lange et al**., [15] | 215 (subgroup Curve<45° at last follow-up) | 31.6±6.1 | 15±6 | 25.1°±8.2° | 29.2°±9.4° | NA | NA | 9.2% |
|  | 32 (Subgroup Curve ≥45° at last follow-up) | 37.6±6.5 | 21±6 | 37.3°±7.0° | 55.0°±8.7° |  |  |  |
| **Montgomery et al**.,[8] | 168 | 33.2 ± 6.5 | NA | 28.0± 10.7 | 33.1± 11.5 | NA | NA | 7.1% |
| **Danielsson and Nachemson** [19] | 109 | 33.2± 9.6 | 24.7± 10.9°* | 29.7± 11.2 | 37.6± 14.7 | NA | NA | NA |
| **Shi et al**.,[17] | 200 | 27.7 ± 5.9 | 21.7 ±7.6* | 30.1± 10.4 | 35.6± 12 | NA | NA | 1% |
| **Cheung et al**. [16] | 144 | 35.5±7.3 | NA | 35 | 43±3 | NA | NA | 6.25% |
| **Korovessis et al**.,[22] | 43 (Subgroup TL) | 29.4±7.1 | 24±6*** | NA | 23.4±7.6 | 8±3 | 9±4 | NA |
|  | (Subgroup L) | 31.2±9.3 | 25±12*** | NA | 25.2±10.5 | 7±4 | 9±5 |  |
|  | (Subgroup T) | 34.2±8.2 | 29±10*** | NA | 31.3±7.2 | 9±3 | 8±3 |  |
| **Cheung et al**., [6] | 98 (Subgroup Improved) | 31±4 | 14*** | 22 | 22 | NA | NA | NA |
|  | 234 (Deterioration Improved) | 31±4 | 20*** | 46 | 46 |  |  |  |
|  | 254 Unchanged | 30±4 | 17*** | 30 | 31 |  |  |  |

* best in-brace, ** intermediate time, ***first in brace
